# Periplasmic Protein Mobility for Extracellular Electron Transport in *Shewanella oneidensis*

**DOI:** 10.3390/microorganisms13051144

**Published:** 2025-05-16

**Authors:** Daobo Li, Xiaodan Zheng, Yonggang Yang, Meiying Xu

**Affiliations:** 1 Guangdong Provincial Key Laboratory of Microbial Culture Collection and Application, State Key Laboratory of Applied Microbiology Southern China, Institute of Microbiology, Guangdong Academy of Sciences, Guangzhou 510070, China; 2 Guangdong Provincial Key Laboratory of Environmental Protection Microbiology and Regional Ecological Security, Guangzhou 510070, China

**Keywords:** microbial respiration, extracellular electron transport, c-type cytochrome, bioelectrochemistry, crosslinking, formaldehyde

## Abstract

Extracellular electron transport (EET) supports the survival of specific microorganisms on the Earth’s surface by facilitating microbial respiration with diverse electron acceptors. A key aspect of EET is the organization of electron relays, i.e., multi-heme c-type cytochromes (MHCs), within the periplasmic space of microbial cells. In this study, we investigated the mobility of periplasmic electron relays in *Shewanella oneidensis* MR-1, a model strain capable of EET, using in vivo protein crosslinking to the MHCs. First, we established that crosslinking efficiency correlates with the spatial proximity and diffusion coefficient of protein molecules through in vitro tests. Based on these findings, we identified distinct molecular behaviors of periplasmic MHCs, showing that the tetraheme flavocytochrome FccA, which also serves as a periplasmic fumarate reductase, forms protein complexes with limited motility, while the small tetraheme c-type cytochrome CctA remains discrete and mobile. Both MHCs contribute to EET for bioelectrochemical nitrate and nitrite reduction. These findings reveal dual mechanisms for organizing periplasmic electron relays in EET, advancing our understanding of microbial extracellular respiration.

## 1. Introduction

Microorganisms on the earth’s surface have evolved diverse metabolic strategies to adapt to heterogeneous physical and chemical conditions in natural environments. For energy metabolism, various natural oxidants such as oxygen, nitrate, oxidized sulfur species, fumarate, and iron/manganese oxides are harnessed as electron acceptors for respiration. Despite the inherent differences in molecular mechanisms required to respire these compounds and the high energetic cost of developing complex respiratory systems, many microbial species demonstrate a remarkable ability to use multiple electron acceptors [1,2]. The dissimilatory metal-reducing microorganism *Shewanella oneidensis* exemplifies this versatility, utilizing oxygen, fumarate, iron(III)/manganese(IV) oxides, nitrate, nitrite, dimethyl sulfoxide, trimethylamine n-oxide, and artificial electrodes as electron acceptors [3]. By employing multiple respiration pathways, these microorganisms thrive across a wide range of redox potentials and survive challenging environmental conditions [2].

Unlike general electron acceptor reduction occurring at the cytoplasmic membrane (CM), the reduction sites for multiple respiration in *S. oneidensis* extend to extracellular space including periplasmic space and the outer membrane (OM). Electron transport across the extracellular space is facilitated by interacting multi-heme *c*-type cytochromes (MHCs) that form electron conduits between the CM and cell surface [4]. The MHCs CctA and FccA (also fumarate reductase), the main electron relays in the periplasmic space of *S. oneidensis*, accept electrons from CM redox components and donate them to either periplasmic reductases (e.g., nitrate reductase NapAB, nitrite reductase NrfA, and the catalytic domain of FccA) or OM cytochrome complexes (MtrABC-OmcA, MtrDEF, and DmsEFAB), which subsequently reduce surface-bound acceptors [5]. While the mechanism of electron transport across the OM has been characterized by resolving the crystalline structure of the MtrABC complex and the interaction between MtrC and OmcA [6,7], understanding electron transport across the periplasmic space remains scant and challenging. Difficulties arise from the subtle organization of MHCs within this space [4,5].

**Figure 1:**
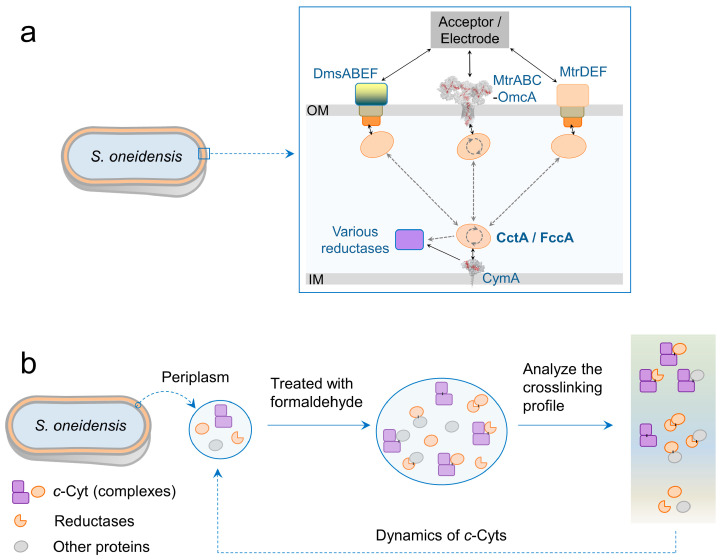
Diagrams on the research contents of this work. (**a**) Cartoon showing the proposed dynamic model of periplasmic electron transport through rolling CctA or FccA. Solid arrows indicate the directions of electron transport. Dashed arrows indicate the directions of protein movement. (**b**) The procedure designed for the in vivo study of periplasmic protein mobility in *S. oneidensis*.

The periplasmic space of Gram-negative bacteria is highly viscous and densely populated with proteins [8,9]. Due to the lack of defined structures for protein anchoring, redox proteins in the periplasmic space are considered to wander and collide randomly with low-diffusion coefficients [5,10,11,12,13]. Although this randomness can restrict long-distance electron transport, *S. oneidensis* actually exhibits high activity levels for extracellular reduction and electricity generation in bioelectrochemical systems [14,15]. Clarifying how FccA and CctA dynamically interact with their redox partners in the periplasmic space is thus essential for understanding the efficient extracellular electron transport (EET). A previous model of periplasmic electron transport proposed, based on the sites of protein interaction determined in vitro, that FccA and CctA roll back and forth between the upstream and downstream redox partners (Figure 1a, [13]). However, this unusual strategy of electronic connection between redox proteins may be a great waste of the capability of electron transport-utilizing MHCs, and to our knowledge, no in vivo studies have substantiated the hypothesis on protein dynamics in the periplasmic space [5,10,16].

In this study, we investigated the in vivo protein dynamics for periplasmic electron transport in the *S. oneidensis* strain MR-1, using the chemical crosslinking of protein–protein interactions with formaldehyde (Figure 1b). Formaldehyde was employed as a crosslinker due to its ability to readily permeate microbial cells and to universally link adjacent proteins [17,18]. The correlation between protein mobility and crosslinking was first examined to establish a methodology for detecting protein dynamics. Next, the molecular weight (MW) analysis of protein complexes from formaldehyde-treated cells was used to investigate the movement of periplasmic MHCs. The MW distribution of FccA and CctA within the crosslinked fractions was tracked to study their specific involvement in chemical crosslinking. Finally, the activity of periplasmic electron transport was characterized using electrochemically active biofilms after protein fixation. Through this research, we distinguished the dynamics of electron relays in the periplasmic space, advancing scientific understanding of extracellular electron transport in *S. oneidensis*.

## 2. Materials and Methods

### 2.1. Organisms and Microbial Growth

The wild type (WT) and Δ*cctA* strains of *S. oneidensis* MR-1 were kindly provided by Dr. Yuanyuan Cheng at Anhui University. For the recombinant expression of 6×His tagged CctA, a strain Δ*cctA*_p*cctA_6×His_* was constructed in this work by transforming the reconstructed *cctA_6×His_* gene into the Δ*cctA*, using pBAD202/D-TOPO as a vector. For the construction of the *cctA_6×His_* gene, a DNA fragment of a 6×His tag (CATCACCATCACCATCAC) was inserted into a site downstream of the *Asp*27 codons of the native *cctA*. All strains were routinely grown in Lysogeny broth (LB) medium at 30 °C. For protein expression of CctA_6×His_, 50 mg/L kanamycin and 1 mM L-arabinose were respectively added at the initial and mid-exponential phases of microbial growth of strain Δ*cctA*_p*cctA_6×His_*. Protein expression was induced for 9 h.

### 2.2. Crosslinking

WT and Δ*cctA*_p*cctA_6×His_* cells were harvested from overnight cultures, washed, and suspended in phosphate buffer (20 mM NaH_2_PO_4_, 80 mM Na_2_HPO_4_, pH 7.4) to an optical density at 600 nm (OD_600_) of 10. Formaldehyde was added to 1% (*w*/*v*), which was followed by the incubation of the suspensions for 10–60 min. For the crosslinking of extracted periplasmic fractions, the incubation time was 5–30 min. Cells for fractionation and biofilms grown on graphite plates were crosslinked for 30 min before further experiments were undertaken. All reactions were performed at 30 °C and quenched with Tris at the end of the formaldehyde treatment.

### 2.3. Fractionation

To extract the periplasmic fractions, crosslinked (CL+) or non-crosslinked (CL−) cells were carefully lysed for 30 min in a phosphate buffer containing 200 mM sucrose and 5 mM Ethylenediaminetetraacetic acid (EDTA). Supernatants were collected by removing protoplasts using centrifugation (8000× *g*, 15 min). Periplasmic fractions were collected by further removing cell debris from the supernatants using ultracentrifugation (100,000× *g*, 1 h).

To extract global soluble proteins and the membrane, cells were lysed in phosphate buffer containing 1 mg/mL phenylmethylsulfonyl fluoride using ultrasonication. Supernatants were collected by removing unbroken cells and large cell debris using centrifugation (8000× *g*, 15 min). A total of 0.2 mM CaCl_2_, 1 mM MgCl_3_, and 10 mg/L DNaseI were then added into the supernatants. Global soluble proteins and the membrane were separated from the supernatants using ultracentrifugation (100,000× *g*, 1 h).

Global soluble proteins produced from the strain Δ*cctA*_p*cctA_6×His_* (CL− or CL+) were flowed through a HisTrap Ni Sepharose HP column (GE Healthcare Bio-Sciences Corp., USA) and washed with the phosphate buffer. The 6×His-tagged CctA (CctA_6×His_)-containing fractions were defined as the column-bound proteins.

Before further experiments, a buffer exchange to Tris buffer (100 mM Tris-HCl, 150 mM NaCl, pH 8.0) was performed for all soluble samples. The membrane fractions were dissolved in Tris buffer containing 1% (*v*/*v*) Triton X-100 for 12 h. Membrane proteins were collected by removing insoluble debris using ultracentrifugation (100,000× *g*, 1 h). Experiments were performed at 4 °C.

Size-exclusion chromatography (SEC) was performed using a home-made column (ø 1 cm, height 90 cm) packed with Superdex 200 PG resin (GE Healthcare Bio-Sciences Corp., Chicago, IL, USA). The buffer conditions comprised using Tris buffer to separate the periplasmic fraction and using Tris buffer containing 0.1% Triton X-100 to separate membrane proteins, at 0.3 mL/min. Absorbance at 409 nm was monitored. Elutes were collected at vials of 0.9 mL. The markers for MW in SEC included thyroglobulin from bovine thyroid (MW 669 kDa), apoferritin from equine spleen (MW 443 kDa), recombinant human IgG (MW 155 kDa), bovine serum albumin (MW 66 kDa), α-chymotrypsin (MW 25 kDa), and CctA_6×His_ (MW 13 kDa).

### 2.4. Sodium Dodecyl Sulfate–Polyacrylamide Gel Electrophoresis (SDS-PAGE) and Heme-Staining

To prepare the samples for SDS-PAGE, microbial cells were lysed at 60 °C in a gel loading buffer (2% sodium dodecyl sulfate, 0.1% bromophenol blue, 10% glycerin, 1% mercaptoethanol in 50 mM Tris, pH 6.8) for 5 min. Protein samples were directly incubated with the gel loading buffer for 5 min at 30 °C. All samples were loaded onto 4–15% gels at normalized protein content. After electrophoresis, *c*-type cytochromes in gels were visualized using general protocol for 3,3′,5,5′-tetramethylbenzidine (TMBZ)/H_2_O_2_ staining. MWs of protein in lanes were evaluated using the software ImageLab V6.1 (Bio-Rad Laboratories Inc., Hercules, CA, USA). The RealBand 3-color Regular Range Protein Marker (Sangon Biotech. Corp., Shanghai, China) was used as an MW standard in SDS-PAGE. Lane profile was exported from Quantity One V4.6.2 (Bio-Rad Laboratories Inc., Hercules, CA, USA).

### 2.5. Fumarate Reductase Assay

Concentrations of FccA in SEC elutes were determined by measuring the intensity of fluorescence produced from the flavin adenine dinucleotide (FAD) cofactor. The FccA molecule was excited at 445 ± 20 nm and emissions at 525 ± 20 nm were recorded. Flavin mononucleotide (FMN) was used as a standard. To determine the enzymatic activity of FccA, vials of SEC elute were individually mixed with 5 mM sodium fumarate and 0.5 mM pre-reduced FMN in Tris buffer under oxygen-free conditions. The absorbance dynamics of the mixtures at 445 nm was immediately monitored. Reduced FMN was prepared by titrating FMN with sodium dithionite in solution. Enzymatic activity was defined to be the maximum reaction rate calculated from the absorbance dynamics. Specific enzymatic activity was calculated by normalizing the protein concentration. The protein concentration was determined using a BCA Protein Assay Kit (Sangon Biotech Co., Shanghai, China) according to the manufacturer’s instructions.

### 2.6. Bioelectrochemistry

Electrochemically active biofilms of WT strain were cultivated on polished graphite plates (4 cm^2^) under potentiostatic conditions. In the electrochemical system, graphite plates served as the working electrode, an Ag/AgCl electrode (KCl saturated) served as the reference electrode, and a platinum wire served as the counter electrode. The working electrode was polarized at +0.2 V versus the reference electrode using a CH1040C Multi-potentiostat (CH Instruments, Shanghai, China). For biofilm formation, LB medium inoculated with cells of the WT strain was initially used as the electrolyte for electrochemical running. The electrolyte was changed to a less-rich medium containing 50 mM lactate and 1 g/L yeast extract after 24 h of running and was thereafter refreshed every 24 h. During the experiment of cyclic voltammetry tests, the electrolyte was replaced with oxygen-free phosphate buffer containing 5 g/L NaCl. Electron acceptors (nitrate or nitrite) were added to 50 mM when needed. Experiments were performed at 30 °C.

## 3. Results

### 3.1. Dependence of Cytochrome Crosslinking on Protein Mobility

Formaldehyde covalently links the protein monomers in a complex or individual protein molecules that swim to close proximity in the periplasmic space [18]. This physical condition aligns with the requirement for inter-protein electron transfer in solution, providing a foundation for probing the dynamics of electron relays in the periplasmic space [19,20]. To observe the effect of molecular collision between periplasmic proteins on crosslinking, the periplasmic fraction of *S. oneidensis* MR-1 was treated with formaldehyde under differentiated protein concentrations and solution viscosities. Specifically, the periplasmic fraction was crosslinked at protein concentrations of 0.8 mg/mL (1× sample) and 4.0 mg/mL (5× sample), where the average molecular distance in the 1× sample was 1.71 times greater than in the 5× sample. Additionally, the periplasmic fraction with 3.3 mg/mL proteins was crosslinked in phosphate buffer containing either 0 M or 1 M sucrose. The addition of 1 M sucrose increased solution viscosity by 3.75 times at 30 °C [21], resulting in a 26.7% decrease in protein diffusion, according to the Stokes–Einstein relation [22]. The crosslinked products were analyzed by monitoring changes in MW using SDS-PAGE and heme-staining.

**Figure 2:**
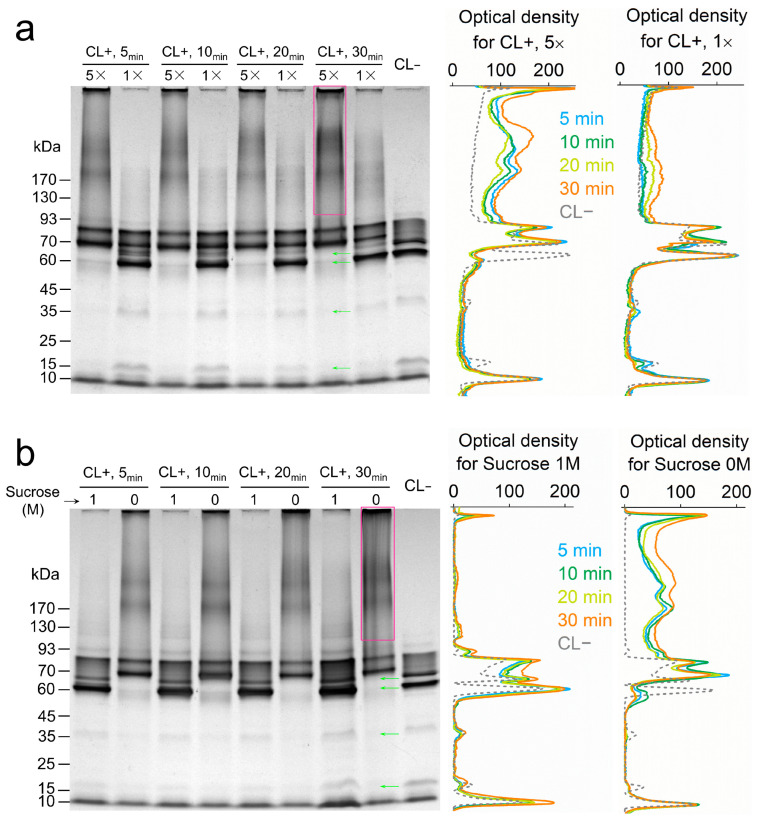
SDS-PAGE and heme-staining results from a formaldehyde-treated periplasmic fraction (**a**) under protein concentrations of 4.0 mg/mL (5×) and 0.8 mg/mL (1×), and (**b**) under different-viscosity solutions (1 M or 0 M sucrose was added). Each lane of gels was loaded with 2 µg protein. CL+: crosslinked; CL−: non crosslinked. The staining intensity of the CL+ lanes was plotted. Rectangles and green arrows mark the produced and vanished bands during crosslinking, respectively. Data are representative of triplicate SDS-PAGE experiments.

Crosslinked cytochrome complexes appeared in gels as a newly emerged band at 70 kDa, band smearing at MW > 100 kDa, and as retentate at the top of the gel (Figure 2a,b). Meanwhile, several bands of native cytochrome at 66 (FccA), 60, 36 (MtrA), and 16 kDa (NapB) disappeared. Non-crosslinked cytochromes showed bands all below 93 kDa, consistent with the MW distribution of periplasmic cytochromes of *S. oneidensis* (Table S1). In the protein concentration-differentiated experiment, crosslinked products in the 5× sample (4.0 mg protein/mL) were clearly visible at 5 min and accumulated until 30 min, based on densitometry analysis (Figure 2a). In contrast, the 1× sample (0.8 mg protein/mL) exhibited only a slight accumulation of crosslinked products until 30 min. The rapid accumulation of crosslinked products was also observed in the 4.2× sample without sucrose, whereas crosslinking was hardly observed with 1 M sucrose added (Figure 2b). These results indicated that cytochrome crosslinking correlates with protein mobility in solution, providing the basis for detecting protein dynamics through in vivo crosslinking of cells.

### 3.2. In Vivo Crosslinking of Periplasmic Cytochromes

**Figure 3:**
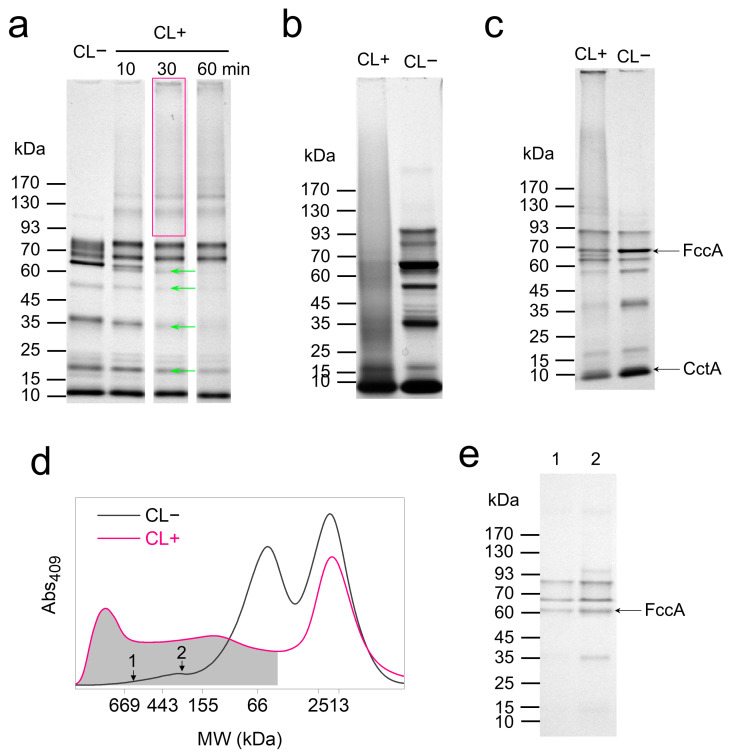
Size distribution of protein complexes after in vivo crosslinking. (**a**) SDS-PAGE of cell lysates of CL− and CL+ (10, 30, and 60 min). Each lane was loaded with 5 µg proteins. Rectangles and green arrows mark the produced and vanished bands during crosslinking, respectively. (**b**) SDS-PAGE results for periplasmic fractions extracted from CL− and CL+ (30 min) cells. (**c**) SDS-PAGE results for total soluble proteins extracted from CL− and CL+ (30 min) cells. (**d**) SEC separation to periplasmic fraction extracted from CL− and CL+ (30 min) cells. Curves are normalized to equal heme content. (**e**) SDS-PAGE results for CL− subfractions marked in (**d**). Data are representative of triplicate SDS-PAGE and SEC experiments.

Based on the results of in vitro crosslinking, wild-type (WT) cells of *S. oneidensis* MR-1 were treated with 1% formaldehyde for 10–60 min to probe the global mobility of periplasmic cytochromes. Cells were lysed, and the MW profile of cytochromes originated from periplasm and the membranes was analyzed using SDS-PAGE and heme-staining. The crosslinked cytochrome complexes in formaldehyde-treated lysates appeared as new bands at 170, 159, 142, and 115 kDa (Figure 3a and Table 1), while bands of native cytochromes at 78, 64 (FccA), 52 (NrfA), 36 (MtrA), and 16 kDa (NapB) disappeared and the band at 11 kDa (CctA) stained weaker. These results indicate the crosslinking of multiple electron relays (FccA and MtrA) and reductases (NrfA and NapB) in the periplasmic space. Bands at 83 and 73 kDa remained unchanged, suggesting the selective involvement of periplasmic cytochromes in in vivo crosslinking. Although the comprehensive crosslinking profile of periplasmic cytochromes can be visualized by analyzing the cell lysates, membrane-bound cytochromes were not excluded and might produce undesired bands.

Further, fraction separation was performed to produce periplasm and membrane components and to specially study the crosslinking of periplasmic proteins. However, the extraction of the periplasmic fraction became difficult after glutaraldehyde treatment (Supplementary discussion and Figure S1), possibly due to protein tethering to membranes or the hindered release of periplasmic protein by the crosslinked OM. The extractable periplasmic fraction of CL+ cells smeared over a wide range of MW above 25 kDa in SDS-PAGE (Figure 3b), with no distinct new bands observed. Considering the possible interference of crosslinking to periplasm extraction, total soluble proteins were extracted via the ultracentrifugation of the cell lysates to remove the proteins anchored in membrane debris. The MW profile of the periplasmic cytochromes in the total soluble proteins was analyzed. As expected, the band profile of the total soluble proteins from CL− cells was similar to that of the extracted periplasmic fraction. Crosslinked cytochrome complexes appeared as bands emerging at 149, 126, 66, and 43 kDa (Figure 3c). Bands at 100, 68 (FccA), 57, 38 (MtrA), 15 (NapB), and 11 kDa (CctA) diminished or vanished after crosslinking. Both these results and the data collected from cell lysates suggested the crosslinking of FccA, MtrA, NapB, and CctA with periplasmic proteins, while NrfA may be crosslinked with membrane proteins.

The MW profile of periplasmic proteins was further analyzed via SEC, which monitor the specific absorbance of *c*-type cytochromes at 409 nm (Figure 3d). In contrast to SDS-PAGE, SEC experiments analyze proteins under non-denaturing conditions, thus preserving native protein interactions. The results revealed substantial crosslinking between periplasmic cytochromes and MW centered at approximately 60 kDa, while the 17 kDa cytochromes showed only slight crosslinking. Additionally, cytochromes of MW > 328 kDa were detected from the CL− sample, exceeding any MWs of monomers of periplasmic cytochromes in *S. oneidensis* MR-1 (Table S1). This result indicates the formation of native FccA complexes or complexes between FccA and other cytochromes. Subsequent SDS-PAGE analysis of marked subfractions identified bands of cytochromes including FccA (Figure 3e).

### 3.3. Differentiated Crosslinking of FccA and CctA

**Figure 4:**
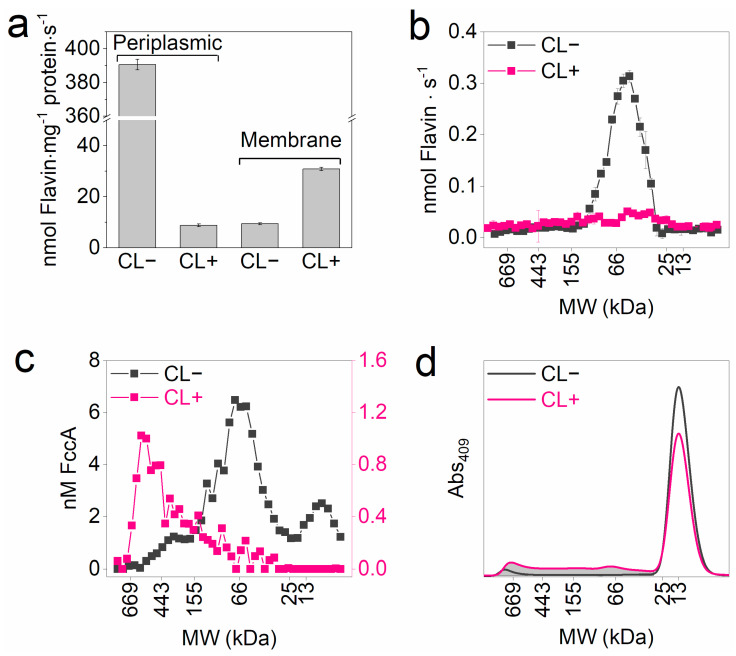
Size distribution of FccA and CctA_6×His_ in crosslinked samples. (**a**) Specific activity of fumarate reductases in periplasmic and membrane fractions. (**b**) Activity of fumarate reductases in subfractions of SEC elutes, Figure 3d. Error bars in (**a**,**b**) indicate the standard error of triplicate assays. (**c**) Concentrations of fumarate reductases in subfractions of SEC elutes, Figure 3d. (**d**) SEC elutes of CctA_6×His_-containing proteins. Duplicate SEC experiments were performed and the results are consistent.

To resolve the in vivo mobility of FccA and CctA in periplasmic space, their MW distribution in the crosslinked sample was characterized. FccA in the samples was traced by analyzing the enzymatic activity for fumarate reduction with reduced FMN as an electron donor and measuring the fluorescence produced from the oxidized FAD cofactor of FccA. The crosslinked periplasm exhibited much weaker activity on fumarate reduction than the native (Figure 4a). In contrast, the membrane fraction of the CL+ sample exhibited a fumarate reduction rate 3.3 times higher than the CL− sample, suggesting the partial membrane binding of periplasmic fumarate reductase during crosslinking. Both fumarate reduction and FAD fluorescence in SEC-eluted periplasmic CL− subfractions centered at 63 kDa (Figure 4b,c). Although activity loss prevented FccA identification in periplasmic CL+ samples, fluorescence analysis revealed FccA-containing subfractions at WM ≥ 150 kDa. This result aligns with the observation in Figure 3 that FccA was extensively crosslinked.

To specifically analyze the MW distribution of CctA, CctA_6×His_-containing fractions were respectively extracted from CL− and CL+ cells of the strain Δ*cctA*_p*cctA_6×His_*. SEC eluting curves of both fractions showed a primary MW peak at 13 kDa (Figure 4d). Higher MW components appeared only in CL+ samples. By integrating the area of the shaded zone, 25.2% of CctA_6×His_ participated in crosslinking. This result is consistent with the observation in Figure 3 that CccA was weakly crosslinked.

### 3.4. Preservation of EET Activity After Crosslinking

**Figure 5:**
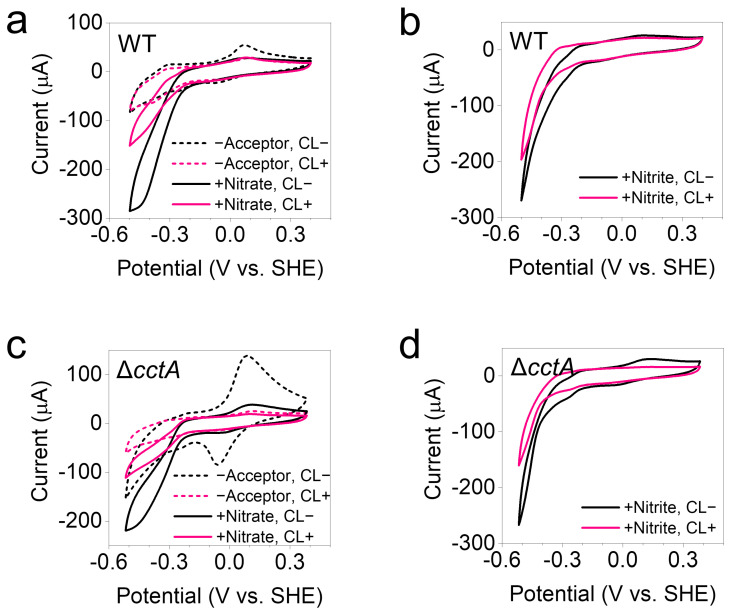
Bioelectrochemical reduction of nitrate and nitrite by crosslinked EET system. (**a**,**b**) Cyclic voltammagrams exhibited the nitrate (**a**) and nitrite (**b**) reduction activities by the WT biofilm before (CL−) and after crosslinking (CL+). –Acceptor: electron acceptor-free conditions. (**c**,**d**) Cyclic voltammagrams exhibited the nitrate (**c**) and nitrite (**d**) reduction activities by the Δ*cctA* biofilm before (CL−) and after crosslinking (CL+). Triplicate biofilms of WT and Δ*cctA* strains were tested and the representative data are shown.

The dependence of EET activity on the protein movement of FccA and CctA was studied in the bioelectrochemical reduction of nitrate and nitrite after crosslinking. Cells were grown on graphite electrodes to prepare electrochemically active biofilms. The bioelectrochemical reduction of nitrate and nitrite by the cells in biofilm electrodes can be achieved via reverse electron transport from the electrode to CymA through periplasmic FccA and CctA [13,23], and then to the periplasmic nitrate/nitrite reductases (Figure 5a). Cyclic voltammagrams characterizing the bioelectrochemical reduction were collected for CL+ and CL− biofilm electrodes. Under the acceptor-free condition, CL− biofilms showed typical redox peaks contributed by extracellular MHCs (Figure 5b,d), indicating a good electronic connection between the MHCs and electrodes. When nitrate or nitrite was added, the electrochemical reduction occurred between −0.21 and −0.50 V (Figure 5b,c). Although crosslinking partially inhibited these reduction processes, 36% and 63% of the native activity was retained, respectively, calculated from current changes at −0.5 V (Figure 5c). Biofilm electrodes of the *cctA* knockout (Δ*cctA*) strain were also cultivated to investigate EET activity under conditions where the free-moving electron relay, CctA, was eliminated during crosslinking. Current changes at −0.5 V showed that Δ*cctA* retained 76% and 99% of the native activity of nitrate and nitrite reduction after crosslinking.

## 4. Discussion

The periplasmic space of *S. oneidensis* has an average width of 23.5 nm [24]. The edge-to-edge lengths of the heme chains in FccA and CctA, determined from their three-dimensional (3D) structures, are 3.5 nm and 4.1 nm, respectively, which are insufficient to form static electron conduits connecting the CM redox proteins to the OMs. Consequently, electron transport across the periplasmic space of *S. oneidensis* likely occur via two mechanisms: (1) the extension of the heme chain through the stacking of several MHCs to form electron conduits or (2) the spatial transport of free electrons by taking the moving MHCs as electron carriers. Uncovering the mobility of MHCs is thus essential to elucidate the electron transport mechanism in periplasmic space. Methodologically, fluorescence microscopy-based particle tracking has enabled the direct observation of protein movement in microbial cells [12,25]. However, resolving nanoscale protein movement within the confined periplasmic space remains technically unfulfilled [11].

Given that physical contact is a requisite condition for electron exchange between molecules of MHCs, in vivo chemical crosslinking provides a meaningful approach to probe protein mobility in cells by universally freezing protein interactions. Using an in vitro formaldehyde treatment for extracted periplasmic cytochromes, we demonstrated that crosslinking efficiency correlates with the spatial proximity of proteins. Specifically, proteins in close native proximity or actively moving into proximity would be more efficiently crosslinked. On this basis, we identified significant differences in the crosslinking behaviors of periplasmic MHCs, particularly FccA and CctA. FccA was mostly crosslinked, as exhibited by the MW analysis of cell lysates (Figure 3a) and specific protein identification from SEC elutes (Figure 4b,c). In contrast, CctA showed a markedly lower proportion of crosslinking. Since the MW of CctA is only one-fifth that of FccA, it is expected to diffuse more rapidly in periplasmic space, according to the Stokes–Einstein Gierer–Wirtz estimation [26]. Thus, the higher degree of crosslinking observed for FccA should originate from its close proximity to other MHCs in its native state. Further evidence of FccA’s binding to other MHCs was provided by the SEC analysis of the CL− samples, where FccA was co-eluted with other MHCs as part of high-MW subfractions (Figure 3d,e). In contrast, CctA appears to exhibit weak binding with other MHCs. Consistent with these findings, NMR studies have shown that FccA has a significantly stronger affinity for MtrA compared to CctA [10,13].

The crosslinking results of FccA and CctA revealed that the organization of periplasmic electron relays differs significantly from previous assumptions. FccA’s binding to other proteins restricts its motility, yet this interaction facilitates the formation of cytochrome clusters, effectively extending the heme chain through interactions with redox partners such as MtrA to support long-distance electron transport. Notably, the retention of nitrate and nitrite reduction activity in bioelectrochemical biofilms of both wild-type (WT) and Δ*cctA* strains after crosslinking demonstrates that immobile FccA can still sustain extracellular electron transport. Such structural organization helps overcome the electron transfer limitations imposed by slow protein diffusion within the periplasmic space [12]. CctA appears to rely on random molecular collisions for transient docking with its redox partners, as shown by the weak crosslinking. The dual modes of electron transport provide alternative conduits for delivering electrons to terminal reductases, which can maintain efficient electron transport either through binding to FccA or through interacting with free CctA [27,28]. This facilitates the extracellular working of multiple reductases with different protein structures, offering flexibility for the molecular processes of respiring diverse electron acceptors and surviving different ecological niches by *S. oneidensis* [29].

In summary, the mobility of the electron relays, primarily FccA and CctA, in the periplasmic space of *S. oneidensis* MR-1 was investigated in vivo, utilizing the correlation between protein proximity and the efficiency of chemical crosslinking. The high levels of crosslinking of FccA and the identification of FccA-containing native complexes indicate the formation of cytochrome clusters involving FccA and its redox partners for electron transport. In contrast, the poor crosslinking observed for CctA indicates its role as an isolated electron relay, relying on protein diffusion and molecular collisions for long-distance electron transport in periplasmic space. These findings reveal a diversified protein organization of periplasmic electron relays to perform extracellular electron transport, offering novel insights into the molecular mechanisms *S. oneidensis* employs to use diverse electron acceptors for extracellular respiration. Moreover, the elucidated dynamics of FccA and CctA in this study provide valuable insights for future efforts to reconstruct the molecular organization of EET systems using advanced imaging techniques such as cryoelectron tomography.

## Figures and Tables

**Table 1:** Cytochrome band changes in SDS-PAGE after crosslinking in Figure 3a.

No.	Source	Band Position (kDa)	Predicted Protein	Band Change
1	Cross-linked	170	-	Emerged
2	Cross-linked	159	OmcA-MtrC	Emerged
3	Cross-linked	143	MtrB-MtrA	Emerged
4	Cross-linked	115	-	Emerged
5	Native	83	OmcA	None
6	Native	78	*Unknown*	Vanished
7	Native	73	MtrC	None
8	Native	64	FccA	Vanished
9	Cross-linked	62	-	Emerged and vanished
10	Native	52	NrfA	Vanished
11	Native	36	MtrA	Vanished
12 and 13	Native	23	SO_4048/CytcB	Weaker
		21		
14	Native	19	CymA	Weaker
15	Native	16	NapB	Vanished
16	Native	11	CctA	Weaker

## Data Availability

The data presented in this study are available on request from the corresponding author.

## Supplementary Materials

The following supporting information can be downloaded at: https://www.mdpi.com/article/10.3390/microorganisms13051144/s1. Supplementary discussion; Figure S1: *c*-Cyt distribution among subcellular fractions of *S. oneidensis* MR-1, WT cells; Table S1: Predicted *c*-Cyts locating into the periplasmic space of *S. oneidensis*.

## Abbreviations

The following abbreviations are used in this manuscript:

CL+	Crosslinked
CL−	Non-crosslinked
CM	Cytoplasmic membrane
EET	Extracellular electron transport
FAD	Flavin adenine dinucleotide
FMN	Flavin mononucleotide
MHCs	Multi-heme c-type cytochromes
MW	Molecular weight
OM	Outer membrane
SDS-PAGE	Sodium dodecyl sulfate-polyacrylamide gel electrophoresis
SEC	Size-exclusion chromatography
WT	Wild-type
