# Supplementary material for: Periplasmic Protein Mobility for Extracellular Electron Transport in Shewanella oneidensis
Source: Microorganisms. 2025 May 16;13(5):1144. doi: 10.3390/microorganisms13051144 (PMC12114092; doi:10.3390/microorganisms13051144)
Supplement: Supplementary file 1 [file microorganisms-13-01144-s001.zip › microorganisms-3563656-supplementary.pdf]

Supplementary Materials

Periplasmic Protein Mobility for Extracellular Electron Transport in *Shewanella oneidensis*

Daobo Li [1,2], Xiaodan Zheng [1,2], Yonggang Yang [1,2] and Meiying Xu [1,2,*]

[1]Guangdong Provincial Key Laboratory of Microbial Culture Collection and Application, State Key Laboratory of Applied Microbiology Southern China, Institute of Microbiology, Guangdong Academy of Sciences, Guangzhou 510070, China

[2]Guangdong Provincial Key Laboratory of Environmental Protection Microbiology and Regional Ecological Security, Guangzhou 510070, China

[*]**Corresponding author**

Meiying Xu, Telephone number: +86 20 87137655, E-mail: xumy@gdim.cn

This PDF file includes:

Supplementary discussion

Fig. S1

Table S1

Supplementary discussion

The UV-Vis absorbance spectra of periplasmic, membrane, and soluble residual proteins (**Figure S1a-c**) fit well with those of typical *c*-type cytochromes (*c*-Cyts), containing a Soret band centered at 408 nm and the *α* and *β* bands centered at 552 and 525 nm, respectively. Based on the absorbance differences at 408 nm and 365 nm, periplasmic *c*-Cyts extracted from crosslinked (CL+) cells were 62.6% in content of those from non-crosslinked (CL-). The lost *c*-Cyts may bind to membrane proteins by covalent link formed using formaldehyde, preventing their detachment from cells during the experiments of periplasmic extraction. Correspondingly, the membrane and soluble residual proteins extracted from CL+ cells contain *c*-Cyts 123% and 176% of those from CL- cells. Besides, release of periplasmic *c*-Cyts from CL+ cells was much slower in kinetics than from CL- cells (**Figure S1d**). These data indicate that periplasmic *c*-Cyts may anchor to proteins or cellular structures during crosslinking and probably lost their motility.

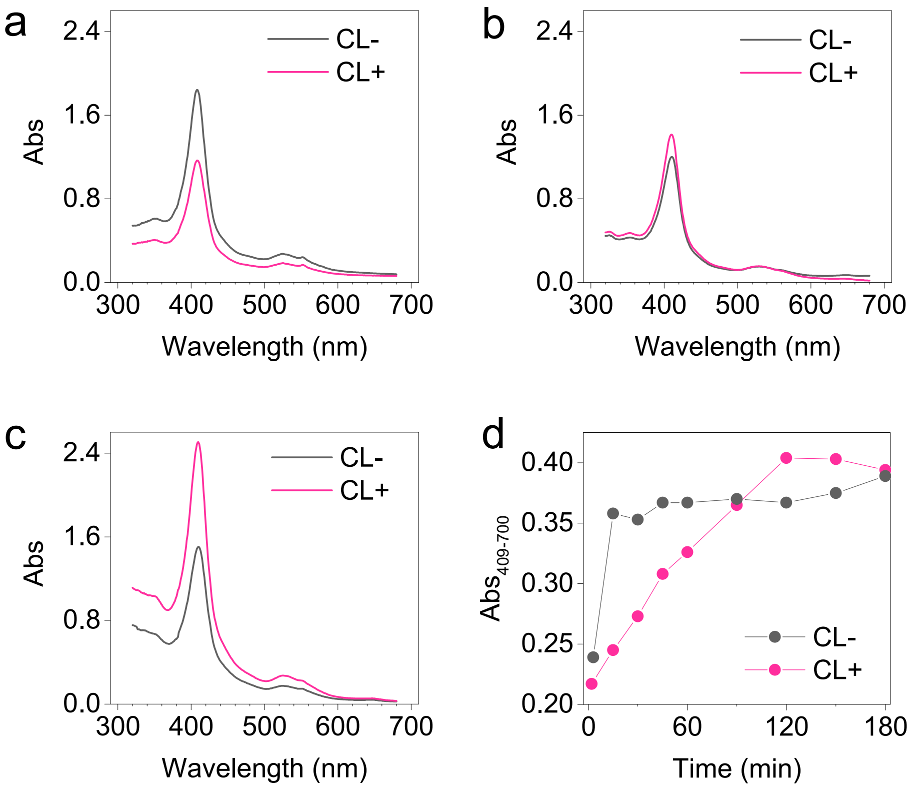

Figure S1 *c*-Cyt distribution among subcellular fractions of *S. oneidensis* MR-1, WT cells. **a**-**c**, UV-Vis absorbance spectra of solutions of extracted (**a**) periplasmic, (**b**) membrane, and (**c**) soluble residual proteins. Crosslinked (CL+) and non-crosslinked (CL-) samples were extracted from equal amount of cells for each fraction. (**d**) Release dynamics of c-Cyts during extraction of periplasmic fraction.

Table S1 Predicted *c*-Cyts locating into the periplasmic space of *S. oneidensis*.

No.	Gene name	Protein	Hemes	MW/kDa	Note
1	*sirA*	Dissimilatory sulfite reductase	8	81.7	
2	*ifcA*	Fumarate reductase	4	63	
3	*fccA*	Fumarate reductase	4	62	
4	*SO_1748*	*c*-Cyt	1	55	
5	*otr*	Tetrathionate reductase	8	54	
6	*nrfA*	Nitrite reductase	5	53	
7	*mtrA*	*c*-Cyt	10	38.6	Bound to OM*
8	*dmsE*	*c*-Cyt	10	38	Bound to OM
9	*mtrD*	*c*-Cyt	10	38	Bound to OM
10	*SO_4360*	MtrA family *c*-Cyt	10	37	
11	*SO_4047*	SoxA-like *c*-Cyt	2	37	
12	*ccpA/bccP*	Cytochrome c5 peroxidase	2	35	
13	*SO_0939*	Split-soret diheme *c*-Cyt	2	27	
14	*SO_4048*	Diheme cytochrome c4	2	21	
15	*cytcB*	Diheme cytochrome c4	2	21	
16	*SO_3420*	*c*-Cyt	1	15	
17	*SO_3300*	Flavocytochrome c heme submit	4	14	
18	*SO_1413*	Flavocytochrome c heme submit	4	14	
19	*SO_3056*	Flavocytochrome c heme submit	4	14	
20	*napB*	Subunit of Nitrate reductase	2	14.5	
21	*shp*	*c*-Cyt	1	14	
22	*SO_3623*	Flavocytochrome c heme submit	4	13	
23	*sorB*	Subunit of sulfite dehydrogenase	1	13	
24	*cctA*	Periplasmic tetraheme *c*-Cyt	4	12	
25	*SO_0717*	Cytochrome c4	1	11	
26	*SO_0714*	Cytochrome c4	1	10	
27	*SO_4142*	*c*-Cyt	1	10	
28	*scyA*	*c*-Cyt	1	9	

*OM, outer membrane.
